# Invasive Research on Non-Human Primates—Time to Turn the Page

**DOI:** 10.3390/ani11102999

**Published:** 2021-10-19

**Authors:** Maria Padrell, Miquel Llorente, Federica Amici

**Affiliations:** 1 Departament de Psicologia, Facultat d’Educació i Psicologia, Universitat de Girona, 17004 Girona, Spain; mariapadrell@gmail.com (M.P.); miguel.llorente@udg.edu (M.L.); 2 Department of Human Behavior, Ecology and Culture, Max Planck Institute for Evolutionary Anthropology, Deutscher Platz 6, D-04103 Leipzig, Germany; 3 Faculty of Life Science, Institute of Biology, University of Leipzig, Talstrasse 33, D-04103 Leipzig, Germany

**Keywords:** welfare, ethics, legal protection, principle of the 3Rs, invasive research ban

## Abstract

**Simple Summary:**

Despite increasing ethical concerns, primates are still often used in invasive research (i.e., laboratory research that causes body manipulations causing them pain or distress and not aimed at directly improving their well-being). Here, we will review previous studies showing that primates have complex behaviour and cognition, and that they suffer long-term consequences after being used in invasive research. We will discuss the ethical problems that invasive research on primates posit, the legal protection that they are, to date, granted in different countries, and summarize the past and current attempts to ban this kind of research on primates. We will conclude why, in our opinion, invasive research on primates should be banned, and non-invasive methods should be considered the only possible approach to the study of primates.

**Abstract:**

Invasive research on primates (i.e., laboratory research that implies body manipulations causing pain or distress that is not aimed to directly improve the individuals’ well-being) has a long history. Although some invasive studies have allowed answering research questions that we could not have addressed with other methods (or at least not as quickly), the use of primates in invasive research also raises ethical concerns. In this review, we will discuss (i) recent advances in the study of primates that show evidence of complex behaviour and cognition, (ii) welfare issues that might arise when using primates in invasive research, (iii) the main ethical issues that have been raised about invasive research on primates, (iv) the legal protection that primates are granted in several countries, with a special focus on the principle of the 3Rs, and (v) previous and current attempts to ban the use of primates in invasive research. Based on this analysis, we suggest that the importance of a research question cannot justify the costs of invasive research on primates, and that non-invasive methods should be considered the only possible approach in the study of primates.

## 1. Introduction

In this study, we will use the term invasive research to refer to all research that (i) is conducted in the lab, (ii) implies stressful and/or painful body manipulations of individuals, and (iii) is not aimed to increase their welfare. Such body manipulations can range from inoculation with infectious agents and surgery to drug testing and blood sampling, but exclude veterinarian applications that directly benefit the animals [1]. According to Directive 2010/63/EU of the European Parliament [2], invasive procedures can have different degrees of severity including (i) non-recovery procedures (i.e., procedures performed under general anaesthesia, at the end of which the animal is killed before recovering consciousness); (ii) mild procedures (i.e., providing animals “short-term mild pain, suffering or distress”, and “no significant impairment of the well-being or general condition of the animals”); (iii) moderate procedures (i.e., causing “short-term moderate pain, suffering or distress, or long-lasting mild pain, suffering or distress” or a “moderate impairment of the well-being or general condition of the animals”), and (iv) severe procedures (i.e., causing “severe pain, suffering or distress, or long-lasting moderate pain, suffering or distress” or a “severe impairment of the well-being or general condition of the animals”). In our definition, we purposely exclude instances of research conducted in zoos, research facilities using observational methods, and research implying body manipulations of wild animals (e.g., trapping) as they posit different ethical concerns that have been extensively addressed elsewhere [3,4]. Here, we will therefore mainly focus on using animals in neuroscience, biomedical and translational research, and other related research areas. We further note here that, although some researchers prefer to refer to the killing of animals at the end of invasive procedures with the term “sacrifice”, which entails a positive connotation (see e.g., [5]), we will stick to the term “killing” throughout the paper because this term conveys, in our opinion, a more objective perspective and is also the term routinely used in legal and technical documents (e.g., [2]). 

Invasive research on non-human primates (hereafter, NHPs) has a long history. Studies on their internal biology date back to Classical times, but the systematic inclusion of NHPs in invasive research only started in the last century [6]. Since then, NHPs have become the object of a variety of studies in disciplines such as medicine, neuroanatomy, genetics, and physiology. Although the relevance and importance of some of these studies has been questioned by some researchers, mostly from research areas other than neuroscience, biomedical, and translational research [7,8], it is undeniable that invasive research has allowed answering research questions that could not have been addressed with other methods, or at least not as effectively [9].

The exact number of NHPs currently used worldwide in invasive research is unknown, but estimates suggest that more than 100,000 NHPs are used every year, mostly in the USA [10]. The Scientific Committee on Health, Environmental and Emerging Risks reported that in 2011, 11 million animals (including approximately 6000 NHPs) were used in scientific procedures in the European Union [11]. In the same report, the European Commission estimated that 8898 procedures were conducted on NHPs in 2014, mostly for the development and safety testing of pharmaceuticals and medical devices, treatment and prevention of infectious diseases, and neuroscience [11]. According to the information reposted by the European Member States, the most frequently used species in invasive research were crab-eating macaques (*Macaca fascicularis*; 80%), marmosets and tamarins (Callitrichidae: 8%), and rhesus monkeys (*Macaca mulatta*; 7%) [11]. More recently, the report on the statistics of the use of animals for scientific purposes in the European Union estimated that 8,235 NHPs were used for the first time for scientific purposes in 2017 [12]. In the United States, the annual report of animal usage informed that 70,797 NHPs were housed in research facilities used in regulated activities; 26,137 of these animals were used in painful procedures and received pain relieving, or similar, drugs, while 802 were subject to painful procedures but did not receive drugs because they would adversely affect results [13]. 

Although NHPs only constitute a small part of all the animals currently used in scientific procedures, it is evident that their use in invasive research is still widespread [10,11]. Understanding whether this is ethically acceptable is therefore a compelling moral imperative for scientists from different research areas. This is even more important if one considers that around 48% of NHP species (including some that are commonly used in invasive procedures, such as crab-eating macaques and several species of marmosets and tamarins) are classified as Threatened by the IUCN. In the following sections, we will discuss which moral statuses and legal protections should be granted to NHPs in line with the most recent scientific developments. To reflect the diversity of the readers of this Journal, we will do that by referring to the current situation of invasive research on NHPs in several areas of the world without restricting our analysis to the European Union case. First, we will summarize recent advances in the study of NHPs that show evidence of highly complex behaviour and cognition that might question their use in invasive research. Second, we will discuss welfare issues that might arise when using NHPs in invasive research. Third, we will review the main ethical issues that have been raised on invasive research on NHPs. Fourth, we will examine the legal protections that are, to date, granted to NHPs in several countries, specifically focusing on the gap between theory and practice. Fifth, we will provide a brief overview of the past and current attempts to ban the use of NHPs in invasive research. In the conclusions, we will discuss why, in our opinion, non-invasive methods should be considered the only possible current approach to the study of NHPs.

## 2. Evidence of Complex Behavioural and Cognitive Life in NHPs

According to some authors, the moral status and the legal protection granted to a species should mainly depend on its behavioural and cognitive complexity [14,15]. Although the great majority of animals experience pain and suffering and are therefore negatively affected by invasive procedures, the additional traits of complex behaviour, emotion, and cognition may increase such suffering beyond ethically acceptable levels. In line with this view, Directive 2010/63/EU of the European Parliament states that the welfare of animals used in invasive research must be improved “by raising the minimum standards for their protection in line with the latest scientific developments” ([2], p. 1). Therefore, understanding the behaviour, emotion, and cognition of NHPs is an essential endeavour to ensure them adequate legal protection by reducing the “pain, suffering, distress and lasting harm” that they experience during invasive procedures [2]. Moreover, new scientific knowledge on behavioural, emotional, and cognitive complexity may support a ban on invasive research on NHPs by, for instance, showing that such complexity does not allow a reduction of pain and suffering below ethically acceptable levels, with full replacement being the only viable option.

To date, NHPs are known to interact in complex ways with their physical environment. Frugivorous monkeys and great apes, for instance, use spatial memory to locate fruit trees in the forest [16,17]. Chimpanzees (*Pan troglodytes*) may retain information on larger fruit trees across seasons [18], recall past events after several years [19], and show exceptional memory skills [20]. Similarly, several monkey species that are widely used in invasive procedures, such as rhesus monkeys and marmosets (*Callithrix jacchus*), form long-term memories of past events that include identity, spatial, and temporal components that suggest the existence of episodic-like memory [21,22]. Several monkey and ape species are also sophisticated tool users; macaques, for instance, can use multiple tools in complex sequences, showing cognitive flexibility and foresight [23]. Chimpanzees are also proficient tool manufacturers, having successfully solved meta-tool tasks that require a flexible and hierarchical organization of behaviour [24,25] and recalled the techniques required to manufacture tools after several years [26]. Similarly, there is evidence that chimpanzees can plan ahead by, for instance, producing and gathering stones that they require for later use [27]. In monkeys, planning abilities may be not as complex, although several species are known to plan one or two steps ahead in sequencing tasks (e.g., rhesus monkeys [28,29] and capuchin monkeys, *Sapajus apella* [30]) and might even anticipate future events (e.g., squirrel monkeys, *Saimiri sciureus* [31]). NHPs also have a complex understanding of the objects surrounding them and partially appreciate causal relationships, although there are likely differences in these abilities across species [32]. 

NHPs also have very complex social lives. Most diurnal NHP species live in large groups that include many females and one to several males [33] who can form long-term social bonds with each other, which are crucial to increasing their fitness (e.g., savannah baboon, *Papio cynocephalus*, and Assamese macaques, *Macaca assamensis* [34,35,36,37,38]). Several NHPs also recognize the social relationships among other group members: monkeys, for instance, know if others have strong social bonds and triadic awareness, understanding the characteristics of social relationships among conspecifics [39,40]. Monkeys can also simultaneously classify group members according to their rank and kinship (e.g., baboons, *Papio hamadryas,* and Japanese macaques, *Macaca fuscata* [41,42]), relying on multiple information sources (e.g., white-faced capuchin monkeys, *Cebus capucinus* [43]). In some NHP species, individuals cooperate with each other by rearing infants together (e.g., Callitrichidae [44]), hunting in group (chimpanzees [45]), sharing food (monkeys and apes [46]), or forming coalitions with specific group members (several monkey and ape species [47,48]). Moreover, monkeys and apes communicate with each other in complex ways, using visual, olfactory, tactile, and auditory signals [49]. For example, some species use specific alarm calls depending on the predator approaching (monkeys and apes [50]), vocalize intentionally (chimpanzees [51]), and even take other group members’ knowledge into account when informing them of the presence of danger (chimpanzees [52]). NHPs are also known to anticipate others’ behaviour and strategically withhold information from group members. Several species, for instance, can account for their conspecifics’ position and visual perspective when competing over food (several monkey and ape species [53,54,55,56,57]). Some species can also anticipate the behaviour of others and understand that the perception/beliefs of other individuals may differ from their own (great apes [58,59]; Japanese macaques [60]). Moreover, several NHP species (including great apes, macaques, and capuchin monkeys) can learn from each other and can transmit this knowledge across generations, giving rise to animal cultures (see [61,62] for reviews). 

NHPs also show complex emotional lives. As compared to other mammals, monkeys and apes have relatively slow life histories with long life spans and late ages at weaning and sexual maturity [63]. Therefore, they spend a long time with their mothers, who are crucial for building affective attachment and promote their healthy socio–emotional development. For monkeys and apes, mothers affect the development of their offspring’s exploration and play style, affiliative and aggressive patterns, and social preferences and future parenting style as well as provide them with opportunities for social learning and ensure their emotional welfare [64,65]. Moreover, both monkeys and apes may show empathic concern and consolation [66]. Chimpanzees and Tonkean macaques (*Macaca tonkeana*), for instance, show affiliative behaviour toward group members that have been the victim of others’ aggression: they preferentially groom conspecifics that have been attacked, especially when they are kin or closely bonded and, in doing so, they decrease the victims’ distress and improve their well-being [67,68]. Some authors have also claimed that NHPs engage in cognitively complex forms of prosociality, spontaneously helping others to reach their goals (chimpanzees [69,70,71]; capuchin monkeys [72]). According to some researchers, NHPs may also show inequity aversion (capuchin monkeys [73]), fairness-related behaviours (several monkey and ape species [74]), and responses to injury and death that closely resemble those shown by humans (e.g., great apes and Sichuan snub-nosed monkeys, *Rhinopithecus roxellana* [75,76,77]). 

The Primate order includes more than 500 species from around 80 genera [78,79], and it is therefore clear that there are important differences across NHP species in terms of the complexities of their cognitive and emotional lives. However, although great apes usually appear to have the most sophisticated abilities, experimental evidence shows that a variety of NHP species “have rich subjective lives filled with intention and emotion” [80] (p. 15). Therefore, these studies provide a view of NHPs as a taxon with highly complex behavioural, cognitive, and socio-emotional lives, long-term memories and a sense of future, diversified social relationships, sensitivity to others’ emotions, empathic concern, and the ability to adopt others’ points of view [66,81,82].

## 3. Welfare Considerations

Some of the welfare concerns that have been raised for NHPs are the same as those associated with the use of other vertebrates in invasive research, including housing in poor environments that do not meet the species’ behavioural necessities, lack of control over the aversive conditions, exposure to painful and stressful procedures, and death [83,84,85]. As for other species, NHPs used in invasive research may also experience additional sources of stress, depending on the protocols used and the severity of the invasive procedures (see e.g., [2]) including transport, social isolation, food and water deprivation, withdrawal from drugs, and repeated surgical procedures [86].

The use of NHPs in invasive research also raises further welfare concerns. Having complex cognitive and emotional skills implies that NHPs may be especially sensitive to the negative consequences of invasive procedures. This might depend on two main reasons. First, NHPs have long-term memories of past events, and some species are also known to plan ahead [19,27]. This implies that they can recall previous stressful experiences for many years, and perhaps imagine their future, amplifying the stress and/or pain they experience. Second, NHPs have very complex social lives. This implies that they have specific needs and requirements that make it very challenging to provide them with decent welfare conditions in a laboratory setting. For example, they typically have long life spans and can thus spend years as experimental subjects [80]; they have complex social relationships that are extremely difficult to reproduce in a laboratory [87]; and they need appropriate environments that promote complex species-typical behaviours and provide sufficient cognitive stimulation [88,89,90]. 

Unfortunately, living conditions in experimental settings hardly satisfy NHPs’ most basic needs. Monkeys up to 15 kg, for instance, can be legally housed in cages as small as 0.5 m^3^ in the USA, 0.63 m^3^ in China, and 3.6 m^3^ in Europe, with metal grid floor being allowed outside Europe [91]. In most countries, NHPs used in invasive procedures are given some form of enrichment activity, such as “objects to manipulate, a variety of foods, […] behavioural training or time in exercise rooms”, and, in some cases, “access to music, television to watch, or touch-screen computers” ([91], p. 4). Although some researchers contend that these forms of enrichment increase NHPs’ welfare (e.g., [91]), such artificial activities provide no ecologically valid experience and no sufficient stimulation from a primatological perspective. Moreover, although “all NHPs are housed with visual and auditory contact with conspecifics” ([91], p. 4), and thus not necessarily in groups, this is certainly not enough to satisfy their complex social needs. Furthermore, depending on the experimental procedures and on the legal requirements of the countries in which NHPs are housed (see below), NHPs may also experience suffering from early maternal separation [92,93,94,95]. In Europe, for instance, macaques are usually weaned around 8 months [91], a developmental stage in which infants are still largely dependent on their mothers under natural conditions [96], whereas transgenic monkeys are usually separated from their mothers from birth (see [91]) with a strong negative impact on their behaviour and physiology [97]. 

Several studies have shown how study subjects that do not undergo death during invasive experiments do suffer long-term psychological effects as a consequence of their traumatic experiences in laboratories [98]. These studies have been mostly conducted on NHPs that have been moved to sanctuaries after spending several years as laboratory subjects and are therefore mostly limited to research on chimpanzees [99]. Traumatic experiences due to invasive research, for instance, are linked to the occurrence of abnormal behaviours similar to posttraumatic stress disorder and depression [100,101]. Traumatized chimpanzees are 19 times more likely to be diagnosed with depression and 88 times more likely to show posttraumatic stress disorder than their wild conspecifics [100]. These problems persist for years, even after animals have been released from laboratories and moved to sanctuaries [98,100]. Although environmental enrichment may reduce the occurrence of some of these negative consequences (e.g., stereotypies and social impairment), it appears to have no effect on more severe forms (e.g., self-harm) [102]. Therefore, invasive research may clearly have a strong impact on NHP welfare, producing negative long-term effects on their physical and physiological well-being.

## 4. Ethical Issues Linked to the Use of NHPs in Invasive Research

The use of NHPs in invasive research is mostly considered necessary to increase our knowledge and improve our health and quality of life [103,104,105,106,107,108,109,110]. By allowing discoveries that are important for humans (e.g., reducing pain or increasing their life expectancy and well-being), invasive research provides humans with benefits that would overcome the costs inflicted to animals in terms of pain, suffering, or death, thus justifying their use (see [14,111] for a discussion). 

From an ethical perspective, the main issue is whether we have the right to inflict pain and death to NHPs if this provides humans with important benefits. Even if NHPs were perfect models to study humans, as most researchers contend [103,104,105,106,107,108,109,110], is causing stress, pain, and/or death to NHPs ethically justified under the premise that human benefits outweigh NHP suffering [112]? For some authors, such a utilitarian approach posits several problems. Its main limit is that it implies an assessment of all the costs and benefits linked to invasive research [113]. First, it is often hard to reliably predict the benefits that a research project really provides, and, indeed, the majority of invasive studies on animals fail to translate to humans [94,111,114]. Second, it is extremely difficult to objectively compare the costs and benefits that involve different species [115,116,117,118,119]. As noted by Arnason [115], benefits to humans often appear to compensate the costs to NHPs only if NHP interests are highly disregarded. Third, quantifying the costs of invasive research to NHPs is no easy endeavour. In theory, all procedures reducing the welfare of NHPs imply some costs, from distress and the impossibility to express the species–specific behavioural repertoire to malnutrition, discomfort, pain, injury, isolation, diseases, and negative valence emotions (e.g., fear, anxiety, and anger), depending on the exact procedures used and their severity (see [120]; see previous sections). These sources of stress can have a strong negative impact on NHP health and welfare, as discussed above [94,121]. 

Even if the costs and benefits of invasive research could be objectively measured, the utilitarian approach may have intrinsic limits because it assumes the potential ethical validity of invasive research as long as the benefits to humans outweigh the costs to NHP (see [94]). Such an approach is not used in human research and has been rightly strongly condemned, both ethically and legally, when having been used in specific historical contexts [122]. Killing or harming human participants (without their consent and providing them with no direct benefits) is ethically and legally unacceptable, no matter how important the benefits for the rest of humanity might be. In other words, human research is based on the principle of deontology, which implies that all humans have an inherent value and are thus considered more than just a means to an end. According to some researchers, there is no reason why the principle of deontology should not be applied also to other species, as any living organism has an inherent value and deserves ethical consideration [123]. Given that species other than humans have a complex perceptual, emotional, and cognitive life (see above), they should also be granted the right not to be harmed or killed for the benefit of others [94]. This approach implies reconsidering the moral statuses of other species in research, i.e., the extent to which NHPs matter for their own sake (see below). Moreover, according to some researchers, additional principles of human research ethics should be applied to NHPs [115], including Beauchamp and Childress’s [124] four principles on autonomy, non-maleficence, beneficence and justice [94], the principles of consent and autonomy [125,126,127], and dissent by the study subjects [128]. For instance, it has been proposed that NHPs should be granted the same legal protection as human subjects who cannot provide informed consent through the assignment of legal guardians and higher safeguards [127].

## 5. Legal Protection Granted to NHPs around the World

Despite recent advances in animal research ethics, the protection that is legally granted to NHPs in research is still largely based on utilitarian principles (i.e., the use of NHPs is allowed if the expected benefits to humans overcome the harm caused to the study subjects). In particular, most national legislations regulating invasive research are currently based on the principle of the 3Rs, which was first formulated by Russell and Burch in 1959 [129]. The principle of the 3Rs pursues (i) the replacement of animals in invasive research through alternative methods requiring no living animals, (ii) the reduction in the number of study subjects involved in research projects, and (iii) the refinement of all the experimental procedures in order to increase animal welfare and reduce their suffering [129]. Historically, the principle of the 3Rs has played a crucial role in drawing attention to the problems of using animals in research and in suggesting possible ways to address them and still underpins the legislation on the protection of research animals in several countries. Below, we discuss four examples (i.e., Europe, USA, Japan, and China) in which the principle of the 3Rs has been differently implemented depending on the pre-existing legal and cultural background. Indeed, there are, to date, no common international regulations for invasive research on NHPs as they exist for research on humans (see [91] for a discussion).

In Europe, the use of NHPs in research is regulated by Directive 2010/63/EU of the European Parliament, which aims to eliminate major disparities in the use of research animals across European countries and ensure a minimum level of protection for study subjects [2]. The Directive in particular rules the replacement and reduction of animals and the refinement of their conditions whenever they are used in “procedures”, i.e., invasive or non-invasive practices for experimental, scientific, or educational purposes that may cause them suffering, pain, distress, or long-term harm [2]. According to Article 4 of the Directive, alternative methods (e.g., cell lines, modelling, or simulations) should be used whenever possible instead of live animals [2]. If there is no alternative method available, researchers should ensure the highest standards of animal welfare and care and reduce the number of animals to a minimum (without compromising the objectives of the project). During procedures, one should reduce pain, suffering, distress, or long-term harm as much as possible, carefully handling the animals, providing high-quality species-specific housing, husbandry, and enrichment activities as well as anaesthesia and pain relief as needed. According to the Directive, the use of NHPs in procedures raises further ethical concerns as it is especially worrying for the general public and requires special care to comply with the behavioural and social needs that NHPs face in captive settings [2]. Hence, procedures on NHPs can be carried out only if there are no alternative methods to reach the same objectives, they are for basic research projects aimed to preserve the species, or if it might have potentially high benefits for humans (e.g., the avoidance, prevention, or treatment of diseases or abnormalities and the development, manufacture, or testing of the effectiveness and safety of drugs, food, and other substances or products) [2]. 

Necessarily, EU Directive 2010/63/EU is a compromise among different interests. On the one hand, for instance, it states that animals have an intrinsic value which must be respected and that they should always be treated as sentient creatures; it states that procedures causing long-term “severe pain, suffering or distress” should not be allowed; and it identifies the full replacement of research animals as its final goal [2]. On the other hand, the EU Directive considers the use of live animals in invasive research to still be necessary [2] and allows NHPs to be routinely harmed and killed during procedures when the results of the project *might* provide enough benefits to humans and no other alternatives are available. More than ten years after its promulgation, indeed, almost 10 million animals are still used every year in scientific procedures in Europe with around 30,000 belonging to “species of particular public concern”, i.e., dogs, cats, and NHPs [12]. 

In the USA, there is a long history of invasive research with little attention to the welfare of animals [130]. This situation started to change in 1985 when several amendments to the Animal Welfare Act (1966) [131], which is the only federal USA law regulating the use of research animals, required the United States Department of Agriculture (USDA) to implement measures ensuring the psychological well-being of NHPs in research [130]. With these amendments, for the first time, research protocols on NHPs must be reviewed by special committees that also include non-scientist members, and researchers must formally check for the possibility of replacing NHPs, reducing the number of animals used, and refining their living conditions by, for instance, ensuring a captive environment that promotes their psychological well-being [131]. However, these amendments still leave research facilities with a high degree of freedom, as they can, for example, develop their own plans to ensure the welfare of study animals. If a research facility receives funding from the Public Health Service (PHS), however, it also must comply with the PHS policy on human care and use of laboratory animals. This policy implies the institution of special committees to review research proposals on animals and to inspect animal facilities as well as requires researchers to comply with the Guide for the Care and Use of Laboratory Animals [132]. According to the Guide, researchers must minimize the distress and pain inflicted to research animals, sedate or anesthetize animals if the procedures cause relevant pain or distress, and provide living conditions that are appropriate for the species and that guarantee health and well-being to the animals [132]. However, the Guide also introduces several exceptions when these exceptions are justified for scientific purposes (e.g., sedation or anaesthesia may be not used during painful or distressful procedures if necessary for reaching the project’s goals) [132]. In May 2019, a spending bill by a committee in the House of Representatives raised serious concerns about animal welfare in research laboratories in the USA and questioned (i) the possibility to really ensure animal well-being and (ii) the validity of animals as models in biomedical research [133]. The bill also urged the National Institutes of Health (NIH) to take concrete steps to reduce and replace NHPs used in invasive research and to regularly report on the progress to Congress [133,134]. In contrast to other NHPs, chimpanzees are offered stronger legal protection in the USA. A study commissioned by the NIH considered the use of chimpanzees for biomedical research inappropriate, especially in consideration of the ethical issues raised by their phylogenetic closeness to humans [135]; so, since 2015, the NIH has completely banned invasive research on chimpanzees [136]. 

Except for the Animal Welfare Act (1966), which sets relatively low standards of protection for research animals [131], all the restrictions to invasive research in the USA only apply to projects funded by the PHS. Moreover, even when researchers must comply with the Guide for the Care and Use of Laboratory Animals [132], several exceptions are granted depending on the aims of the study. For instance, if researchers aim to specifically test for the effects of social deprivation on NHPs, the obligation of ensuring the social well-being of the study subjects does not have to be fulfilled [130]. In our opinion, if laws seriously ensured welfare standards for NHPs in invasive research, studies on social deprivation should simply not be allowed because they do not comply with these standards. This introduces a certain degree of freedom in the application of the rules that should ensure the welfare of research animals. Indeed, even if the Guide suggests that NHPs should be housed in social groups [132], in 2007, only 38% of the monkeys in indoor labs shared their cage with at least one other individual [130,137]. Moreover, researchers have often noted how abnormal behaviour that signals severe stress in NHPs (e.g., self-harm behaviour) has not decreased since the introduction of the Animal Welfare Act [138], suggesting that the measures taken so far are far from sufficient to really ensure the “psychological well-being” of NHPs. Despite these problems, in 2000, the NIH still recommended an *increase* in the number of NHPs used in invasive research as they would be “crucial” for biomedical and behavioural research and, indeed, according to the USDA, the number of NHPs used for research reached 75,825 in 2017, an increase of 22% as compared to in 2015 [133]. 

In Japan, the use of animals in research is regulated by the Law for the Humane Treatment and Management of Animals (Law No. 105, 1973; see [139]). According to this law, researchers should avoid purposelessly killing or inflicting harm and injuries to animals, and they should treat them “properly” [139]. Following an amendment in 2005, the Law further requires researchers to implement the principle of the 3Rs, reducing and replacing research animals when possible and refining their living conditions [139]. For research animals, the Law further refers to the Standards Relating to the Care and Management of Experimental Animals [140]. The Standards consider the use of animals in invasive research necessary but also foster the implementation of the 3Rs [139,141]. In 2006, the Science Council of Japan (2006) published the Guidelines for Proper Conduct of Animals Experiments, which provides some very general advices on the aspects that researchers should consider when conducting research on animals [142]. The Guidelines, for instance, attribute the responsibility of properly conducting animal experiments to the directors of the research institutions and suggest that researchers should take into account animal welfare and reduce the pain and distress caused to the study subjects as allowed by their research aims [142]. 

Crucially, all these restrictions are not legally binding, but they are “promoted appropriately with the understanding of the people” ([142], p. 1). Research institutions, for instance, should voluntarily establish internal regulations on animal research that reflect the general principles expressed in the Law, the Standards, and the Guidelines, but there is no obligation to doing that, and there is extensive freedom in how these standards are effectively implemented in different laboratories. Similarly, there is no obligation to report the number of NHPs used for research purposes [143]. Therefore, animal research in Japan appears to generally prioritize the researchers’ right to academic freedom over the animals’ rights to health and well-being. For some authors, Japanese legislation on invasive research is surprisingly permissive and, although this may depend on the religious and cultural peculiarities of the country [141,142], it is also true that part of Japanese society actually demands more stringent rules and transparency in the use of research animals by, for instance, claiming access to experimental protocols on NHPs and other animals (see [143]). 

In China, the Ministry of Science and Technology (MOST) is responsible for regulating the use of NHPs and other animals in invasive research, in line with the Statute on the Administration of Laboratory Animal Use approved by the State Council in 1988 [144,145]. In 2006, the MOST also issued the Guideline on the Humane Treatment of Laboratory Animals [146], which requires animal research facilities to orient their practices to the principles of the 3Rs and animal welfare and to institute specific committees to review and control the ethics of research projects requiring the use of animals [144,145]. The Guideline, for instance, requires researchers to inflict as little pain as possible during their experiments and to consider the species’ welfare when housing animals [146]. However, the Guideline only provides very general recommendations and, being mere regulations of the MOST (and not laws), failure to respect them implies lower sanctions as compared to what happens in other countries [147] (see [144]). Through time, these Statutes and Guidelines have been implemented and complemented through a variety of regional, provincial, and municipal laws, guidelines, and policies [144,145], leading to a complex interplay of different layers that leave a certain flexibility to animal research facilities [147,148]. The Provincial Department of Science and Technology, for instance, is responsible for the use of research animals at the provincial level, but local Administration Offices of Laboratory Animal Use enforce the regulations and provide licenses to both the researchers and research institutions that breed or use animals for research [145].

The Chinese case is especially relevant for the international implications it has. Biomedical research is crucial for the Chinese economy and it is continuously expanding [145,149,150]. In 2011, China had around 40 authorized NHP breeding centres employed more than 100,000 people in animal research labs, used around 25,000 NHPs, and exported 25,000 more to other institutions in Europe and the USA (see [145]). Given that Western countries usually have a much stricter legislative system on invasive research and higher standards for animal welfare, several researchers and companies in Western countries have outsourced animal testing in China, or they have moved (or threatened to move) to China to conduct their studies [147,151]. In this way, researchers face lower legal standards and can conduct studies they would not be allowed to run in Europe or the USA [149,152]. Recently, for instance, the neuroscientist Nikos Logothetis left his position as a director of the Max Planck Institute for Biological Cybernetics in Germany (where he conducted invasive research on NHPs to mainly study the neural mechanisms of perception) to co-direct a new International Center for Primate Brain Research in Shanghai, which will house around 6,000 NHPs, including transgenic monkeys [150]. In 2014, videos of lame and bleeding monkeys secretly recorded in his lab led to an official investigation; although Logotethis and his colleagues were relieved from all accusations and charges at the end, public pressure and growing skepticism on invasive research led the neuroscientist and his colleagues to move their research groups to China [150]. Similarly, after the 2019 USA spending bill raised concerns about animal welfare in research laboratories (see above), the president of the National Association for Biomedical Research, Matthew Bailey, expressed his concern about the interference of Congress and stated that, in this way, “research will be more likely to move to other countries” [134]. 

These cases have fuelled the debate on the consequences of increasing the protection of research animals. For some people, increasing the legal protection of NHPs and other animals might lead scientists to simply leave for countries with lower standards of animal welfare [11,153,154]. This reasoning echoes economic discussions on the need to lower the protection of workers in Western countries to make them more competitive on the global market and avoid the delocalization of industries and other economic activities. However, a race to the bottom is no answer to the risks implicit in having different welfare standards across the world. Increasing the protection of NHPs is an ethical question that goes beyond merely practical considerations, and that concerns the scientific community and the general public alike (see [153]). Moving (or menacing to move) to another country to conduct invasive research and thus circumvent national legislation has ethical implications that cannot be ignored [147] and that should lead to practical consequences (see below). 

## 6. Brief Overview of Attempts to Stop Invasive Research on NHPs

To date, pressure to increase the legal protection of NHPs and to drastically reduce or ban their use in invasive research mainly comes from three different sources: (i) campaigns or petitions by animal organizations with support from public opinion; (ii) parliamentary questions and spending bills by political groups within national institutions; and (iii) scientific publications by authors who are often experts in ethics, philosophy, or alternative methods.

Campaigns by animal organizations are mainly aimed to inform the public about the state of the art of NHP research and raise concern about the current practices. Following a long-running campaign by Cruelty Free International, for instance, most airlines have stopped transporting NHPs for use in invasive research [155]. Moreover, animal organizations often rely on groups of lawyers that can initiate legal actions to protect specific animals. The Animal Legal Defense Fund, for instance, recently filed two lawsuits against the USDA for its failure to protect the psychological well-being of primates used in biomedical research (New England Anti-Vivisection Society vs. Goldentyer [156]) and for inconsistent inspections of NHP research facilities (Animal Legal Defense Fund and Rise for Animals vs. USDA [157]). Similarly, in 2015 the “Stop Vivisection” movement, with a document signed by 1.17 million citizens, demanded that the European Commission abrogate Directive 2010/63/EU on the protection of animals used in invasive research and ban their use in Europe. The European Commission reiterated that, “for the time being, animal experimentation remains important for protecting human and animal health, and for maintaining an intact environment”, but it also stated that Europe is “working towards the ultimate goal of full replacement of animals” ([158], p. 10). Although these campaigns often fail to reach the ultimate goal of banning invasive research, they engage public opinion and maintain high pressure on institutions to raise welfare standards for research animals. 

Parliamentary questions and spending bills by political groups within national institutions have a similar function. Often, they are proposed by institutional minorities and have little chance to really achieve their goals. The spending bill approved by a committee in the USA House of Representatives in 2019, for instance, strongly urged research institutions in the USA to more effectively reduce and replace NHPs in invasive research and to report on their progress to Congress. However, the final version of the bill negotiated by the Senate and the House of Representatives was much duller, recognizing the importance of NHPs in invasive research and moderating the requests advanced [134]. Still, these initiatives contributed to raising awareness in the public and making researchers feel that they are accountable for their practices. In 2019, the Environmental Protection Agency of the USA announced the goal of a reduction of invasive studies on mammals by 30% by 2025 and its complete ban by 2035 [134]. Similarly, the National Institutes of Health (NIH) in the USA has organized two workshops on NHP research in the last four years, including one on the science and ethics of biomedical research [134]. Although 2035 is a far-off date, and invasive studies will still be allowed pending administrators’ approval despite the lack of real advances following the NIH workshops (see [134]), these events force researchers to at least justify their use of NHPs and explore alternatives. 

Although most researchers consider invasive research as still being necessary [103,104,105,106,107,108,109,110], there are several scientific groups and research centres specifically working on alternatives to animal testing. These include the Center for Alternatives to Animal Testing at the Johns Hopkins University in the USA and the University of Konstanz in Germany, which support the creation, validation, and use of alternative approaches to animal research, education, and product-safety testing and publish the peer-reviewed journal ALTEX, which is focused on alternatives to animal experiments. Other research centres include the European Union Reference Laboratory for alternatives to animal testing in Ispra, Italy, which coordinates and implements validation studies of alternative methods, and the non-profit organization Americans for Medical Advancement, which opposes the view that animals are valid models on which to conduct research and fosters the use of alternative scientific procedures. Moreover, there is also a long tradition of studies authored by experts in ethics or philosophy clearly positioned against the use of NHPs in invasive research [94,115,159,160]. These studies often call for the need to overcome the principle of the 3Rs and recognize a higher moral status for NHPs. Some researchers, for instance, demand that NHPs be granted fundamental rights, such as the right to life and bodily and mental integrity, and that these rights be defended before a court through legal representatives [159]. 

The positions of primatologists, anthropologists, comparative psychologists, and other experts on NHP behaviour and cognition (hereafter referred to as “NHP experts”) with regards to the use of NHPs in invasive research is often not clear. In some cases, NHP experts have been consulted during the preparation of reports or opinions on the use of NHPs in invasive research, but their role has usually been very marginal [130,131]. The Weatherall’s report from 2006 [8] and the review by Bateson and colleagues from 2011 [7], for instance, are some of the most famous reports on the use of NHPs, but there are virtually no NHP experts among their authors. The Weatherall’s report was written by a working group consisting of experts “drawn from outside the active non-human primate research community”, in the own authors’ words ([8], p. 15). In the Bateson review, eight out of the nine panel members were neuroscientists, physicists, or professors in medical areas with no experience in NHP behaviour, and the chairman of the panel was Patrick Bateson, an eminent behavioural biologist with vast knowledge in several research areas but little experience with important ethological, behavioural, and cognitive aspects of NHPs [7] (also see [161]). 

To our knowledge, there is also no international working group led by NHP experts aimed at banning the use of NHPs in invasive research. The only notable exception is perhaps the Great Ape Project, an international movement of ethicists, anthropologists, and primatologists who call for the extension to great apes of fundamental human rights (i.e., right to life, freedom, and non-torture [162]). Despite its limit to focus only on great apes, which can be largely justified by the limited knowledge we had on other NHPs at the time the movement started, the Great Ape Project crucially moves beyond the principle of the 3Rs, fostering the recognition of a higher moral status for great apes. Other projects have had a similar focus on chimpanzees and have resulted in a series of positive changes in terms of legal protection granted to this species, at least in some countries. In Japan, for instance, two organizations, SAGA and GAIN, have obtained the complete ban of invasive procedures on chimpanzees, thanks to the extensive support of the public and the active involvement of primatologists [163]. These organizations foster the post-mortem use of chimpanzees and the use of other non-invasive procedures, and they are currently keeping a database of all the apes in Japan that are used in invasive procedures in order to gradually extend them the protection now granted to chimpanzees [163]. Moreover, these organizations have helped establish a sanctuary for laboratory chimpanzees dismissed from biomedical research, in which chimpanzees are provided with life-long care in social groups and more natural settings [164,165,166]. Similarly, chimpanzees in the USA are better protected than other NHP species; although there is no federal law prohibiting the ownership of chimpanzees, their use in invasive procedures has been completely banned since 2015, and they are individually registered in a database to better monitor their welfare [136,167,168].

## 7. Conclusions and Future Perspectives—Beyond the 3Rs

To date, abundant experimental evidence has shown the complexity of the social, emotional, and cognitive lives of monkeys and apes and the long-term effects that invasive research can have on their physical and physiological well-being [169,170,171]. These scientific advances clearly show that invasive research implies huge costs for NHPs, and therefore raise crucial ethical issues on the use of NHPs in invasive research. Still, the legal protection of NHPs is generally inspired by the principle of the 3Rs, although there is a lot of variation across countries in the practical implementation of this principle. Currently, the pressure to improve NHP legal protection is mainly exerted by animal organizations supported by public opinion, political groups within national institutions, or researchers working on ethics, philosophy, or alternative methods. These often call for a switch from a utilitarian approach (and the principle of the 3Rs) to a deontological approach that questions the general validity of NHP research from an ethical perspective [14,84]. 

Despite claims by several institutions around the world on the long-term goal of replacing NHPs in invasive research (e.g., [2]), the situation for NHPs has not changed much. Actually, NHPs are still abundantly used worldwide in invasive research, and the refinement measures that are generally used cannot ensure, in our opinion, the welfare of animals with such complex socio-emotional and cognitive lives. Therefore, despite the benefits that invasive research on NHPs may have for humans (including their use as models to test preventive and therapeutic strategies during pandemics), we argue that their use in invasive procedures should be immediately banned. The European Union establishes the full replacement of research animals as its final goal [2], but it also identifies the reasons why the fulfilment of this goal might still require time, including “the risk aversive nature of society” (which would be resistant to new alternative methods), the lack of funds to develop and validate alternative research methods, and “factors related to scientific practice and career progression where dynamics such as competition, the reputation and track record of researchers […] and entrenchment discourage switching from NHPs to alternative […] models” ([11], p. 21). These issues, however, can be easily addressed. As scientists, we can drive the change by ensuring that resources are preferentially devoted to projects using alternative methods, asking researchers to publish more precise details when invasive procedures are used, urging journal editors to ask the opinions of primatologists concerning welfare aspects during the review process, and/or otherwise encouraging researchers to conduct non-invasive studies. By massively diverting research funds to non-invasive research, for instance, it would be possible to foster new research approaches that might emerge to be as beneficial for humans as is invasive research. As NHP experts, we can use experimental evidence to contribute concerted legal opinions and reports worldwide and develop a concrete institutional plan with clear timelines to successfully ban invasive research on NHPs. 

Overall, we are convinced that ethical and scientific reasons call for the immediate ban of NHPs in invasive research. Such an approach calls for an overcoming of the principle of the 3Rs; after more than six decades from its original formulation, we think it is time to move forward and re-discuss our current goals based on the competencies and knowledge we have acquired after all these years. Reducing the number of NHPs and refining their living conditions are measures that cannot adequately guarantee their welfare and should thus be considered ethically inappropriate. NHPs need to be granted life and health in the first place, and these rights cannot be waived for research purposes no matter how beneficial it might be for humans. This is even more important when considering that most NHP species are currently threatened with extinction [172]. We are responsible for preserving biodiversity and protecting natural habitats, whereas they play a key role as ecosystem engineers [173]. Based on the advances in the study of NHP behaviour and cognition, and on ethical considerations, we believe that the time has come to definitively stop non-invasive research on NHPs, and that non-invasive behavioural and experimental methods should be considered the only possible approach to the study of NHPs.

## Data Availability

Not applicable (review).
